# How to develop young physical activity leaders? A Delphi study

**DOI:** 10.1371/journal.pone.0286920

**Published:** 2023-09-29

**Authors:** Isobel P. Steward, Emma S. Young, Sufyan Abid Dogra, Elizabeth Stamp, Andy Daly-Smith, Kammy Siddique, Kelly Morgan, Jamie Crowther, Jennifer Hall, on behalf of the JU:MP research & development team

**Affiliations:** 1 Bradford Institute for Health Research, Bradford Teaching Hospitals NHS Foundation Trust, Bradford Royal Infirmary, Bradford, United Kingdom; 2 Faculties of Life Sciences and Health Studies, University of Bradford, Bradford, United Kingdom; 3 School of Sport, Exercise and Health Sciences, Loughborough University, Loughborough, United Kingdom; 4 Centre for Applied Education Research, Wolfson Centre for Applied Health Research, Bradford Royal Infirmary, Bradford, West Yorkshire, United Kingdom; 5 Centre for Development, Evaluation, Complexity and Implementation in Public Health Improvement (DECIPHer), Cardiff University, Cardiff, United Kingdom; Lahore Medical and Dental College, PAKISTAN

## Abstract

The International Society for Physical Activity and Health advocates for increased capability of the physical activity workforce as a key ingredient to a system-based approach. Young leader programmes are gaining traction globally as peers are a primary influence on young people and positive role models are important for increasing or maintaining physical activity. Yet, there is limited understanding of ‘what works’ for training young physical activity leaders. This study aims to develop a consensus on how to identify and support young people to become physical activity leaders. An iterative three-phased mixed methods Delphi consensus approach. A rapid review focused on the feasibility, acceptability and impact of existing young leader physical activity training (phase one); focus groups (n = 3) and interviews (n = 6) with 15 practitioners and young leaders to examine young physical activity leader training needs (phase two); and a three-round questionnaire process (phase three). Stakeholders (n = 43) from across the public, voluntary and education sectors, academics and young leaders completed all questionnaires. A consensus was reached for 75 statements related to: young leader traits prior to and following training, recruitment methods, training content, delivery format and context, relationships, incentives, and skill development. The Delphi process, combining insight from multi-sectoral stakeholders, identified a range of factors that underpin young leader training programmes. These factors should be applied to develop a curriculum and comprehensive training programme to provide young leaders with the required capability to be effective within their roles, and ultimately support an increase in physical activity amongst children and young people.

## Introduction

Over half (55.4%) of children aged 5–16 years in 2020–2021 were not being physically active for the promotion of health and development i.e. meeting guidelines of 60 minutes of moderate-to-vigorous physical activity per day [1]. Physical activity has traditionally been defined as “any bodily movement produced by skeletal muscles that results in energy expenditure” [2] however, a more holistic conceptualisation considers the culturally specific spaces and contexts in which this movement occurs and influencing factors, such as interests, emotions and relationships [3]. Physical activity levels reduce with age; the transition to adolescence is a key time when physical activity starts to decline [4]. Whilst UK guidelines for recommended minutes of physical activity are significantly lower for adults (16+) compared to children, at 150 minutes across the week, 33% of young adults did not meet these guidelines in 2020–21 [5]. This is a key public health issue as physical inactivity is associated with poorer health and wellbeing in children and adults, including increased prevalence of anxiety, depressive symptoms, obesity, cardio-vascular disease and social problems [6–9]. Physical inactivity, globally, is estimated to cost over $68 billion USD/year [10].

The International Society for Physical Activity and Health (ISPAH) advocacy document “Eight Investments that Work for Physical Activity”, outlines key priorities to address physical inactivity [11]. Sitting under the investment “Sport and Recreation for All”, increasing the capacity of organisations to deliver physical activity through the physical activity workforce, and the utilisation of positive role models, are considered key facilitators for inspiring and encouraging population level physical activity [11]. Young leaders are individuals aged between 16–25 years who engage with others to encourage a target behavior (e.g. physical activity) [12]. Training young people to become physical activity leaders is a promising solution to address the challenge of inactivity in children and young people [13] through diversifying and upskilling the physical activity workforce.

Positive role models have been identified as important for encouraging behaviour change during childhood, related to the combination of exposure to active people and social support [14]. There is evidence that children favour role models that are relatable [15] and as such, younger adults are preferred role models [16]. Additionally, the influence of peers is strongest in adolescence and early adulthood than at any other point in the life course, shaping attitudes, values and behavior significantly [17]. Crucially then and owing to their relatability, young leaders have a unique opportunity to encourage and support physical activity amongst children and young people. There is evidence that utilizing young people to encourage a change in their peers’ behaviours is associated with effectiveness across health, education and social spheres, including tackling youth violence [18], smoking cessation [19] and benefitting academic achievement [20]. In terms of physical activity, a recent scoping review of peer-led interventions identified 43 studies [13]. Findings highlighted mixed intervention effects regarding physical activity amongst children and young people aged 8 to 22 years receiving the peer-led interventions with some interventions showing promise. However, the authors reported that the majority of interventions involved young leaders delivering a set intervention designed by adults, rather than being trained and supported to be a physical activity leader in a more independent capacity [13]. This approach is symptomatic of hegemonic socio-political ideals that ‘adults know best’ which undermine the ideals of youth leadership [21].

Harnessing young leaders to tackle physical inactivity amongst children and young people can also serve as a mechanism for positive youth development of the leaders themselves through support and training [22]. This ‘dual benefit’ of young leadership has been evidenced in leadership contexts including physical activity, education and drug and alcohol addiction, whereby young leaders have reported outcomes including increased positive health behaviours, self-determination, self-esteem, knowledge-building skills and employability [23–28].

Despite the promise of investing in young people to become physical activity leaders [13, 22], there is a dearth of information surrounding the processes underpinning recruitment and training of young leaders, and the contextual factors influencing the impact of such training on young leader capability and confidence to deliver physical activity. The research question the present study aims to address is: how can young people be identified and supported to become physical activity leaders, and what are the key desired outcome(s) of young leader training programmes? Our primary research objective is to conduct a Delphi study to develop a consensus on how to develop young physical activity leaders.

## Materials and methods

### Study design

**Fig 1:**
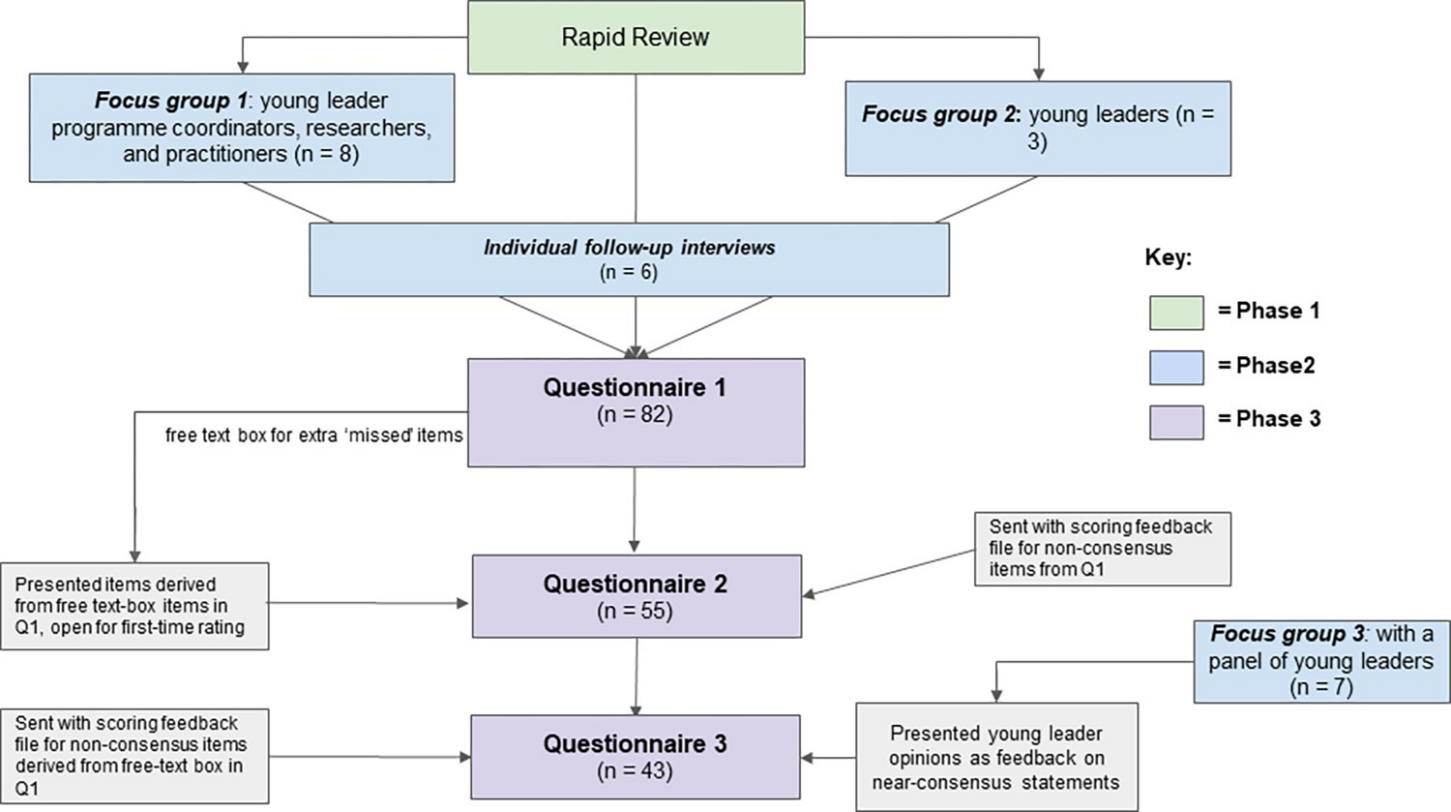
Three-phase study design.

A traditional Delphi consensus is defined as a group communication process to achieve a consensus of opinion around a specific topic [29]. A Delphi approach was chosen for consensus building given the inbuilt flexibility and opportunity for reconsideration and reflection amongst expert stakeholders [30]. For this study, a modified Delphi process [31] including three iterative phases was used to reach a consensus on how to identify and support young people to become physical activity leaders: 1) a rapid review of literature, 2) qualitative consultations with practitioners and young leaders (three focus groups and six interviews), and 3) a three-round questionnaire process; Fig 1. Traditionally, Delphi studies employ paper based questionnaires, with three distinct stages: 1) initial ‘idea generation’ (via an open questionnaire), 2) initial rating of items generated in first questionnaire; and 3) rerating of items that do not reach consensus at initial rating- this can be repeated a number of times. The current study modified the traditional Delphi method by facilitating the study online rather than through paper based questionnaires, and replacing the initial ‘idea generation’ questionnaire with data from focus groups and interviews conducted with a core section of the Delphi panel. The current study also conducted a rapid review of literature focused on the feasibility, acceptability and impact of existing young leader training programmes to contribute to the ‘idea generation’ phase of the research, allowing the literature to be identified quickly whilst maintaining some of the rigour of a systematic review. Replacing the initial idea generation questionnaire with a literature review is a widely-accepted modification to the traditional Delphi method [32], and rapid reviews are commonly adopted as part of Delphi approaches [33–35]. Consultation with a small subgroup of the Delphi panel, to inform questionnaire content, has also been utilised as a method in other Delphi studies [36]. Ethical approval was obtained from the University of Bradford (REF: E877, 20/04/21).

### Rapid review

The rapid review addressed three questions: 1) What methods are effective in developing young physical activity leaders? 2) What components of young leader training programmes are feasible and acceptable? and 3) What are the impacts, and mechanisms of impact, of training packages on the young leaders? An initial search of the literature (October 2020) identified a scoping review exploring the rationales and effects of peer-led physical activity interventions involving young people through qualitative and/or quantitative methods [13]. Given the relevance of the topic area, our search strategy involved screening the studies included in the Christensen scoping review [13], alongside replicating their search for the time-period November 2017—December 2020. We searched five databases (Embase, PubMed, Scopus, SPORTDiscus, Web of Science) using the search terms (‘children’ OR ‘young people’) AND (‘peer-led’ OR ‘young leader’) AND ‘Physical activity’, to replicate the search criteria in the original paper to ensure a rigorous process was maintained as much as possible throughout. Inclusion criteria were as follows:

- English-language papers published from 2010 onwards
- Young people aged 16–25 years in peer and/or young leader roles
- Report on the feasibility, acceptability and/or impact of the young leader training through qualitative and/or quantitative methods

**Fig 2:**
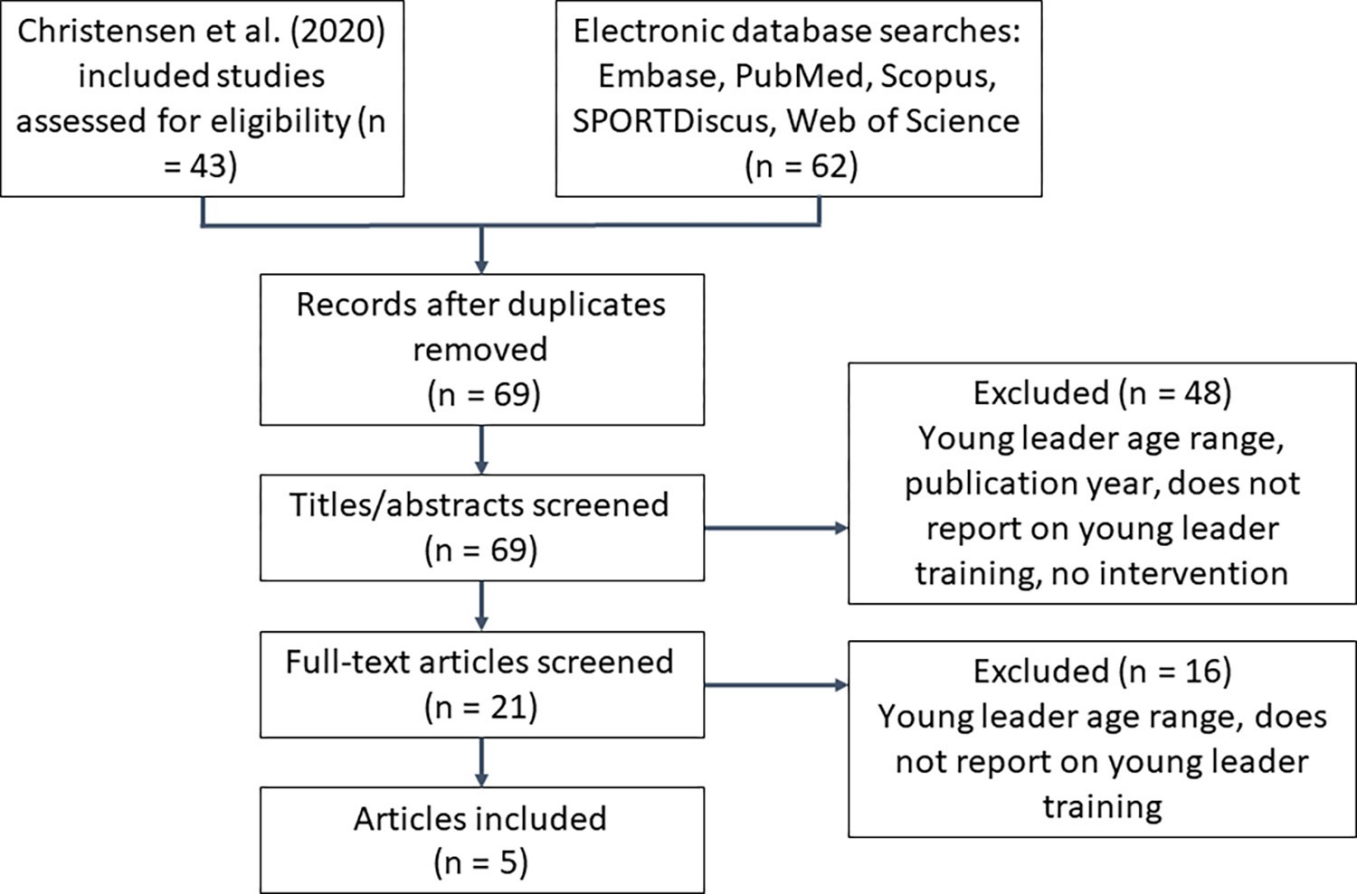
Rapid review flow diagram.

There were no exclusion criteria. Five papers from the original scoping review reference list were identified as potentially relevant. The database search returned 62 studies including 36 duplicates, resulting in 26 unique studies. One researcher (IS) screened all unique studies (n = 31). A second researcher (JH) screened 40% of the studies and there was full agreement regarding inclusion; see Fig 2 for an overview of the review process. Data extracted from the studies included: age of young leaders, the setting, and any information from the methods or findings related to young leader training/development. A thematic synthesis was conducted using NVivo 12.0 (QSR International Inc., MA, USA). The extracted data was coded deductively based on pre-specified categories related to the research questions; including training features, impact, acceptability, effectiveness and feasibility. Definitions of these terms can be found in Table 1. Coding was conducted by one researcher (IS) and reviewed by another researcher (JH) and any ideas or disagreements were shared through discussion and subsequent revisions to codes were made.

**Table 1:** Definitions of codes used in the qualitative data synthesis.

Code	Definition
Training Features	Aspects of the intervention’s young leader training
Impact	Any other effect the training and the role of a young leader had on the participants
Acceptability	How well the training was received by the young leaders
Feasibility	How easy the training is/was to deliver

The Mixed Methods Appraisal Tool (MMAT) [37] was used to assess the quality of the studies included in the review. This involved a screening process, answering ‘yes’, ‘no’ or ‘can’t tell’ to a series of questions in the five categories of qualitative research, randomised controlled trials, non-randomised studies, quantitative descriptive studies and mixed methods studies. In adherence with the recommendations from the MMAT manual, a single score for each paper was not collated; instead a narrative summary [38] was developed.

### Qualitative consultations

Building on the rapid review findings, two online focus groups were conducted with young leader and practitioner experts in April 2021, to explore experiences of young leader training programme design, delivery and receipt, following which an initial questionnaire was drafted. Six focus group participants were invited back in May 2021 for follow-up interviews, to review draft initial questionnaire items and allow for revisions and additions. After the second Delphi questionnaire findings were collated, a third focus group was conducted with young leaders in September 2021. The information collected in this final focus group informed the development of the final Delphi questionnaire.

#### Qualitative consultation—recruitment and participants

Participants were sampled purposively based on existing relationships and knowledge of local experts related to young physical activity leader development. We aimed to recruit young leaders (focus group 1) and practitioners involved in the design / delivery of young physical activity leader training (focus group 2) from the local area. After the initial 2 focus groups, a sub-sample of these participants, identified through convenience sampling, were invited to participate in individual follow-up interviews. Some young leader participants in the third focus group were identified through their participation in focus group 1, and other young leaders were identified through contacts with local young leader training programmes. For all qualitative data collection, practitioner participants were contacted by the research team via email, and young leader participants were contacted via practitioner partners. All participants returned signed consent forms prior to study participation as well as providing verbal consent at the start of each focus group and/or interview.

#### Qualitative consultation methods

The initial two focus groups were designed to explore the characteristics and traits of young physical activity leaders and training methods needed for developing young leaders; the findings of the rapid review were presented to facilitate discussion alongside more open-ended discussion. Follow-up interviews aimed to consolidate the findings and capture any additional items through asking for their views on the initial draft questionnaire, in order to finalise the questionnaire. Thus, the initial draft questionnaire, developed from the rapid review and focus group findings, formed the basis of the interview guide. Interviewees were asked for their general views about the statements, and whether anything was missing, unclear, or required expansion. The third focus group, which was conducted in between questionnaire rounds two and three, aimed to: a) inform the final questionnaire round and b) bolster the contribution of young leaders’ voices, through including only young leaders in the focus group and incorporating a summary of their responses into the final questionnaire. Within the focus group, participants were presented with near-consensus (i.e. 65–75%) statements for in-depth discussion. Data were transcribed by one researcher (IS or EY). Qualitative data were analysed thematically by two researchers (IS and EY). All focus groups and interviews took place over Zoom due to COVID-19 restrictions at the time of data collection.

### Delphi questionnaires

The rapid review and qualitative consultations served as the initial ‘idea generation’ phase, which informed questionnaire development. Three iterative questionnaire rounds were implemented to develop a consensus related to important factors underpinning young physical activity leader training.

#### Delphi consensus—Recruitment and participants

Participants were recruited on the basis of them being involved in the design, delivery or evaluation of young sport or physical activity leader programmes, conducting research related to physical activity and young leadership, and/or lived experience of being a young sports or physical activity leader. Purposive and snowball sampling methods were used to recruit participants, by searching relevant websites (for example, Active Partnerships and Universities), affiliations with relevant institutions/organisations such as Active Partnerships and community organisations, and approaching mutual contacts of members of the core research and wider team to request recommendations from their networks. Qualitative consultation participants were also invited to participate in this phase. Participants were provided with an information sheet when initially contacted by email, which also included a personalised link to the first questionnaire for them to complete, should they wish. Participants were required to provide informed consent at the start of each questionnaire round. Participants provided their role in the first questionnaire, and were thereby stratified as: academics; education, public, or voluntary sector stakeholders; or young leaders. Academic stakeholders included those in research and/or University lecturing roles, whereas educational stakeholders worked in a school (up to 18 years) setting.

#### Delphi consensus—Data collection

Sub-topics from qualitative consultations were used to construct questionnaire headings and items: 1) Characteristics and traits of young physical activity leaders, 2a) Recruitment, 2b) Training content, 2c) Format and context, 2d) Incentives and rewards, 2e) Relationship building, and 2f) Skills development. In each questionnaire, participants were given an opportunity to rate their level of agreement with each statement via closed, likert-scale questions, ranging from 1 (strongly agree) to 9 (strongly disagree). A 9-point likert scale was adopted as it readily permits classification of responses as negative agreement (score of 1–3), neutral (score of 4–6) or positive agreement (score of 7–9), and it is sensitive enough to capture true evaluations. As such, 9-point likert-scales are often used in Delphi studies [32]. Opportunity for re-rating was provided in subsequent questionnaires should items not reach the threshold for consensus, to ensure participants were afforded the opportunity to reflect and alter their scoring on these items. For these items, participants were provided with their previous scoring alongside the average score of the whole sample and their stakeholder group (questionnaires two and three) and summary views of the young leaders that took part in focus group three (questionnaire three only). In the first questionnaire, there was also an open text box for participants to add any other considerations they deem important, not currently included in the questionnaire. These considerations were then incorporated into questionnaire two. Questionnaire two was sent only to participants that completed questionnaire one, and questionnaire three was sent only to participants that completed questionnaire two. See Table 2 for an overview of the timing and content of each questionnaire. Full copies of the questionnaires used in this study can be found in S1–S3 Files. Questionnaires were administered online via Smart Survey and each participant received a secure, personalized access link. Participants were given three weeks to respond to each round, and were sent a reminder email one week prior to the deadline if they had not completed the questionnaire at that point.

**Table 2:** Content and timings of the Delphi questionnaires.

	Questionnaire 1	Questionnaire 2	Questionnaire 3
Timing	June 2021	August 2021	October 2021
Statements from rapid review and qualitative consultations	Initial rating of level of agreement with statements	Re-rate statements that did not reach agreement in Q1	Re-rate near consensus statements based on focus group 3 findings
Information provided	n/a	Controlled feedback file that documented (a) % agreement across the entire sample (b) mean score according to the participant’s stakeholder group, and (c) a reminder of the participants previous response for these statements	A brief narrative on focus group findings relating to these statements
Statements from open text box	Open text box idea generation	Initial rating of level of agreement with statements	Re-rate statements that did not reach agreement in Q2
Information provided	n/a	n/a	Controlled feedback file that documented (a) % agreement across the entire sample (b) mean score according to the participant’s stakeholder group, and (c) a reminder of the participants previous response for these statements

#### Delphi consensus—Data analysis

Analysis of questionnaire data was completed using Microsoft Excel and involved calculating central tendencies (mean, median, and mode), agreement percentages and interquartile ranges following each questionnaire round, to inform the next questionnaire. For each statement, values were calculated for the whole sample as well as for each stakeholder group (academic, public sector, voluntary sector, education sector, young leader). Consensus was defined as an agreement percentage of above 75%, meaning that 75% of participants scored an item 7, 8 or 9 (positive agreement), or 1, 2 or 3 (negative agreement) [32]. Any items that met this threshold were not included in subsequent questionnaires and (where the agreement was positive) were included in the consensus statement. Items that had been in both questionnaire round 1 and 2, and for which less than 65% of participants in questionnaire 2 scored the item a 7, 8 or 9, were discarded at this point. Items that were near positive consensus (agreement percentage of 65–75%) were the focus of the third focus group and revised in the third questionnaire. Any item that had not reached a consensus after the third questionnaire was not included in the toolkit.

## Results

### Rapid review

Five papers were included in the review; see Table 3. Two studies were completed in the United Kingdom [39, 40], 2 in Australia [41, 42] and 1 in the USA [43]. Young leaders ranged from ages 14–29 years with the majority being secondary school age. All interventions were school or university-based, except one community-based [40].

**Table 3:** Characteristics of the 5 papers included in the rapid review.

Lead author	Year	Intervention name	Setting (Country)	Population: Young leaders	Training format
Corder	2016	GoActive	School (UK)	Students in year 9 and above (13–16 years old)	1 hour session plus continued support by the study team and intervention facilitators
Foley	2017	Students As Lifestyle Activists (SALSA)	School (Australia)	Year 10 students (15–16 years old)	1 day workshop led by university students
Jenkinson	2012	Girls! Lead! Achieve! Mentor! Activate! (GLAMA)	School (Australia)	Year 10 girls (15–16 years old)	1 day workshop led by a teacher in the school
Khan	2011	Nutrition and Exercise for a Healthy Living University Module	University (USA)	Senior level students (over 18 years old)	1 hour a week for 8 weeks led by graduate students
Taylor	2016	Girls on the Move Leadership Programme	Community (UK)	Young women (14–29 years)	Variety of training options varying from an intensive 5 day course to courses spanning over an 8–10 week period led by external course leaders

#### Quality assessment

Most papers had clear research questions and collected relevant data. All studies used mixed-methods. For the quantitative element, 1 study was classified as a randomised controlled trial [39] and was moderately scored. The remaining studies [40–43] were labelled as non-randomised controlled trials and scored highly. For the qualitative element, 2 papers [41, 43] scored low due to limited explanation of the approach used. See S1 Table for full MMAT results.

#### Training methods

Training varied in content and intensity across studies. Duration of training varied from one day [39, 41, 42] to eight weeks [43], with one study giving young leaders control over how long their training lasted [40]; see Table 2. The training was delivered by researchers [39, 42, 43], volunteer university students who received prior training [41] or external course leaders [40]. Training focused on the content of the intervention, as well as developing and refining leadership skills. Three training programmes incorporated group work through discussions and partner activities [39, 40, 43] and two provided young leaders with qualifications or certificates for completing the training [40, 41].

#### Impact on young leaders

Overall, training adequately prepared young leaders to deliver their role. 94% of the young leaders in GoActive reported improved leadership skills [39] and all young leaders in GLAMA (Girls! Lead! Achieve! Mentor! Activate!) [42] reported feeling confident to lead following training. The young leaders also described developing leadership skills applicable to other aspects of their lives (e.g., future employment) and 7 of the 8 young leaders continued their leadership in the following academic year [42]. Course graduates delivering activities in the community increased from 6% to 15% following ‘Girls on the Move’, with some young leaders continuing to deliver physical activity sessions 2–3 years following training [40]. In the university-based study, the training facilitated positive attitudes around leading the intervention, however the young leaders did not feel they had the knowledge to increase physical activity among children and other young people following the training [43]. Many of the young leaders suggested incorporating more content-specific activity, and public speaking and teaching practice into the training [43].

#### Acceptability

Acceptability of training varied across studies. While 88% of the young leaders in the GoActive study found the training ‘fun’, they reported comprehension issues [39]. In the SALSA (Students As LifeStyle Activists) program, 91% of young leaders stated they would recommend the experience to their peers and felt the training was relevant, age-appropriate and engaging [41]. Acceptability was low in the GLAMA study [42], as young leaders were reluctant to miss lessons, work experience or after-school clubs to attend training [42]. 97% of young leaders in ‘Girls on the Move’ reported that the training met their expectations regarding knowledge and skills development, however, the training did not adequately prepare them for unsupervised session leadership [40]. The university-based study involved young leaders contributing to developing the content of the programme that they would be leading, which facilitated acceptability [43].

#### Feasibility

Feasibility of the training methods was similar across school-based studies [39, 41–43]. Researchers had difficulties engaging young leaders due to competing educational priorities and institutional constraints [39–42], and there were communication issues between schools and research teams [39]. Training that was low intensity and low-cost was more feasible to deliver within a school context [41]. Training delivery within the ‘Girls on the Move’ community-based programme was reported as feasible by tutors [41].

### Qualitative consultations and questionnaires

**Fig 3:**
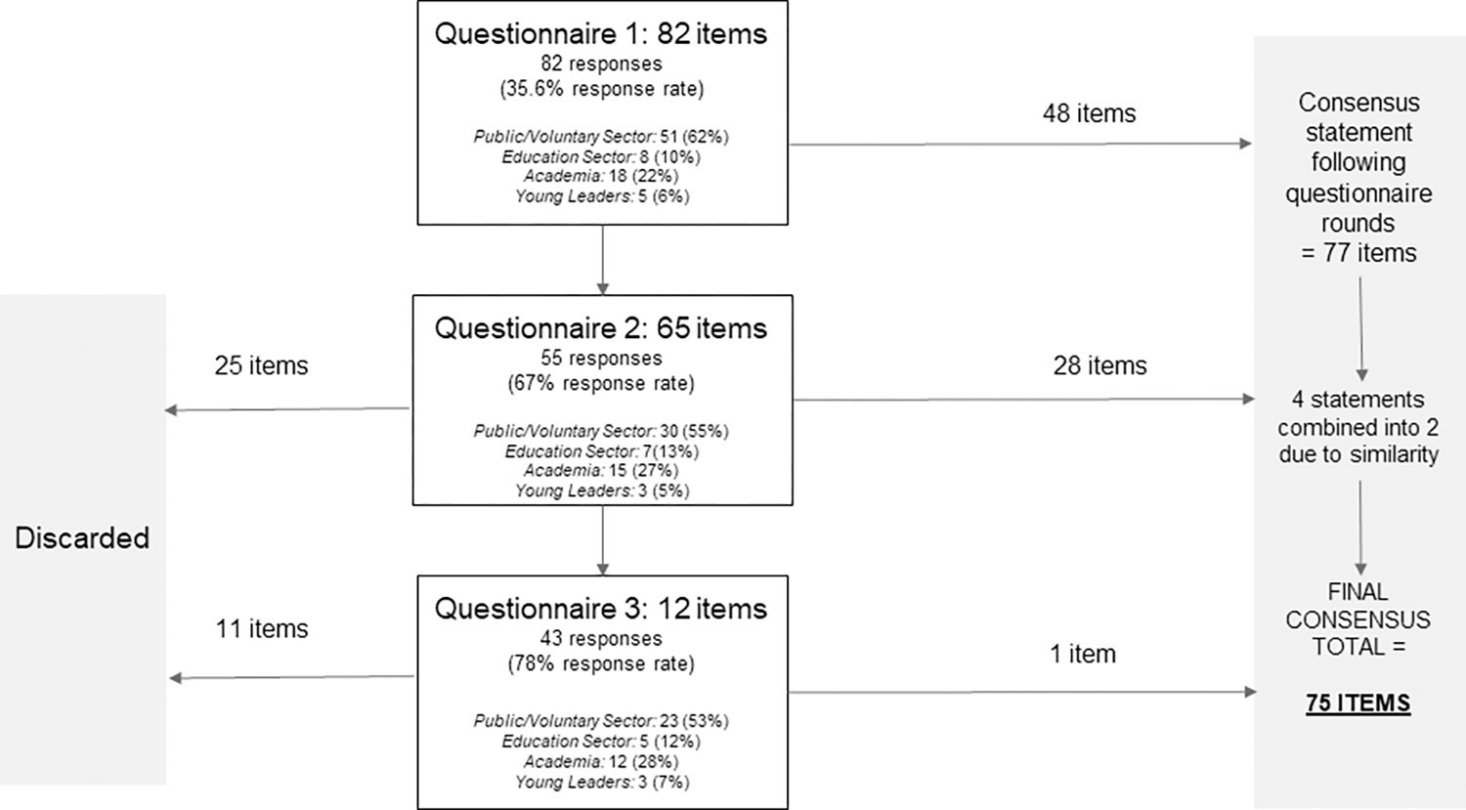
Questionnaire and Delphi consensus statement flow diagram.

The focus groups comprised 11 experts; including three young physical activity leaders, two researchers, and six practitioners all currently involved in the delivery of young physical activity leader training. The follow-up interviews were conducted with three young leaders and four practitioners. Then, follow-up interviews were conducted with three young leaders and four practitioners. Levels of participation across the questionnaires, and the number of items that reached consensus in each round, are displayed in Fig 3. The third focus group, conducted between questionnaire two and three, included seven young leaders. Key findings from the various stages of the Delphi process are provided below.

#### Focus groups 1 and 2

There was much discussion relating to timings, settings, content and context of training within both the young leader and practitioner focus groups. Practitioners indicated that training should take place over an “extended period of time” in order to meaningfully develop skills and confidence of young leaders and build relationships, and both groups felt that training should include a mixture of delivered content (including accredited qualifications), one-to-one mentoring and support, and ‘on the job’ training including shadowing of experienced leaders and delivery. A stakeholder with a background in youth work suggested that “creative methods” typical in youth work settings are more appropriate than “classroom based methods” to engage young people in training and also ‘model’ how to work with children and young people to engage them in physical activity. However, the participants also emphasised the importance of flexibility, including tailoring the training programme to the needs of the individual young people. Young leader participants agreed that young physical activity leaders have a responsibility to reach the least active, and to do this it is important to think about physical activity holistically rather than a narrow focus on sport, and to have an understanding of the barriers to physical activity within the community in which they will be working; as such, they thought that training programmes should develop understanding around what constitutes physical activity and methods and approaches for consultation with the community:

“*we need to teach them about assets*, *and community development skills… they’ll start to understand then why are you doing training around hyperlocal communities*, *co-design*, *community engagement*, *we need to give them those softer skills*, *even as a leader*, *as a sports leader*, *for them to understand”*

When discussing requirements and characteristics of training delivery staff, both young leaders and practitioners voiced that trainers should deliver in a way that makes the young people feel supported and listened to by them, and emphasised the value of a positive relationship between trainers and young leaders. The young leader participants also commented that this relationship may be facilitated by trainers being older, but relatively close in age to the young leaders, so that they are someone that they can “look up to” (i.e. a role model) but also feel sufficiently relatable. In a similar vein, practitioners spoke of the value of recruiting young people from diverse backgrounds who represent communities, to support the development of relatable physical activity role models for children and young people. There was agreement across both focus groups that the most important characteristic of young people for developing effective physical activity leaders is their drive and enthusiasm to “make a difference”. These findings were used to develop a draft first questionnaire, containing 64 items.

#### Follow-up interviews

Follow-up interviews provided more in-depth insights that were used to refine the first questionnaire; categories were revised alongside changes to the wording of nine items. For example, interview narratives revealed that young leader traits may hold differing importance depending on the temporal context in which they are framed, which led to the separation of ‘characteristics and traits’ into ‘before training’ and ‘after training’ categories within the initial questionnaire. It was felt that some traits may not be required from the outset as they can be developed through the training process; confidence was highlighted in this regard by multiple interviewees. Interview accounts also identified points of nuance that helped to ‘break down’ items; an additional 18 items were added to the questionnaire. For example, interviewees voiced that it may be more important to have a deep knowledge and understanding of the local area in which young leaders are working, than being ‘from’ that area, and that whilst a positive overall experience of training is desirable, it may also be beneficial to “push young leaders out of their comfort zone” which is likely to elicit some transient negative emotions.

#### Questionnaire 1

Fourty-eight of the 82 items included in questionnaire 1 achieved positive consensus in the first round. Eleven of these items received a score of 7–5 from over 95% of respondents:

- Training should give young leaders the opportunity to practice leading sessions
- During training, young leaders should develop: (i) the ability to motivate others, (ii) communication skills, (iii) team-working skills
- Once a young leader has completed their training they should be: (i) enthusiastic, (ii) willing to be involved, (iii) trustworthy, (iiii) honest, (v) responsible, (vi) patient, and (vii) punctual.

No items received negative consensus and so no items were discarded at this point. Open text responses were provided by 12 questionnaire respondents. Across these responses, various participants referred to the importance of tailoring training to the context of both the individual young leaders and the wider local community, and emphasised the value of supporting disengaged young people to become leaders in line with a positive youth development approach:

“*We need to develop a pathway of opportunities for young leaders to dip in & out of along their journey—one size does not fit all so we should deliver a varied offer to make it inclusive to all young leaders”*

“*Given that different contexts vary so widely*, *it’s very important that any training remains culturally appropriate*, *but also tailored to different communities*. *I’d suggest that any training would benefit from having a connection to the community”*

“*I have had a contentious relationship with schools as opportunities to be young leaders tend to be given to the same young people as it is seen as a reward*, *when really those young people who don’t conform to school norms can flourish in informal situations when given a new power dynamic…*. *we can’t rely on the young people brave enough to stick their hand up and volunteer*, *it’s up the adults and facilitators to inspire disengaged young people into something new”*

There were also various more specific suggestions raised for incorporation into the next questionnaire by individual respondents, such as the training supporting the development of young leaders’ organisational and behavioural management skills, and resilience and empathy as young leader traits/characteristics. An additional 31 items were added to questionnaire two based on the open-text responses.

#### Questionnaire 2

Twenty-eight items achieved positive consensus in the second round. Two of these items received a score of 7–9 from over 95% of respondents: prior to training young leaders should be willing to learn new skills, and young leaders should develop skills during training to ensure sessions they deliver as a leader are inclusive for all involved. Whilst no items achieved negative consensus across all participants, 25 items were discarded as they did not achieve near positive consensus following the opportunity to re-score.

#### Focus group 3

The focus group involved discussion of nine near consensus items. For near-consensus items concerning ‘pre-training’ characteristics, the discussion focused on whether characteristics could be developed, in particular within the timescales of a young leadership programme. The young leaders agreed that empathy was a necessary prerequisite as this could take significant time to develop, but resilience and being physically active should not be pre-requisites as they could feasibly be developed through training. The young leaders expressed mixed views regarding the necessity of including sport-specific skills development in training, with some voicing that this should be tailored to the future ambitions of the young people in terms of any particular sports or physical activities they would like to specialise in. When considering whether training should be delivered by people from local organisations, the young leaders expressed that the nature of the organisation with regards to locality was inconsequential with regards to the training content, but that such contact with local organisations could help them build networks that would be beneficial for future leadership opportunities. The young leaders felt that offering formal qualifications as part of young leader training is important as it can (i) be a motivator for getting young people on the training course, and (ii) set young people up for future employment. The young leaders indicated that they themselves would not have joined their course if it did not offer formal qualifications.

#### Questionnaire 3

Based on the analysis of questionnaire three data, one further item achieved positive consensus: offering formal qualifications as part of young leader training programmes. Eleven items that did not achieve positive consensus (or negative consensus) were discarded.

### Developing young physical activity leaders: Consensus statement

**Fig 4:**
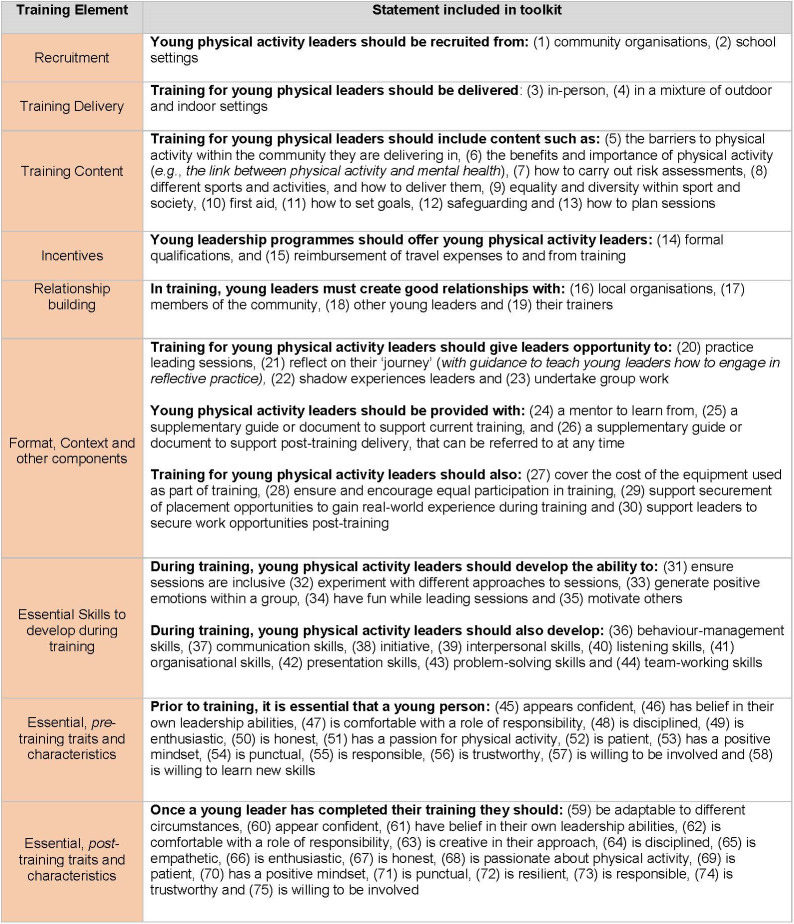
Statements that reached positive consensus regarding young physical activity leader development.

The final consensus includes 75 statements that experts agreed are important for developing young physical activity leaders, under the headings: young leader traits prior to training (n = 14), recruitment methods (n = 2), training content (n = 9), training delivery (n = 2) training format and context (n = 14), relationships (n = 4), incentives (n = 2), skills to develop during training (n = 14) and young leader traits following training (n = 17). See Fig 4 for all statements.

## Discussion

The primary objective of this research was to develop a consensus on how to develop young physical activity leaders, including ways to identify and support young people and the key desired outcome(s) of young leader training programmes. The research objective was met by delivering a rigorous three-phase multi-method Delphi process. A rapid review and qualitative consultations examining the feasibility, acceptability and impact of existing training programmes informed the development of the questionnaire phase of the Delphi study, which involved expert stakeholders from across research and practice. There is a dearth of existing literature examining young physical activity leader training approaches; this study presents original, practice-based evidence. A consensus was reached for 75 statements of factors that are important for developing young physical activity leaders, which can inform the evidence-based development of young physical activity leader programmes.

The study highlighted key considerations for developing young physical activity leader training, including the importance of group work, which reached 95% consensus. Wider literature evidences benefits of group work in various settings [44, 45]. Leadership development courses are, in general, limited by curriculums using individual-focused methods, which do not replicate the real life contexts in which leaders operate [46]. The rapid review indicated shorter training packages were more feasible to integrate into young leaders’ lives, but were less likely to equip them with the necessary skills and experience, and giving young leaders flexibility regarding training duration was found to be acceptable [40]. Focus group data revealed that whilst flexibility was important, this should be balanced with some set timings to build structure. The statement ‘giving the young leader flexibility to fit the training into their timetables’ was close to, but did not reach a consensus. Involving stakeholders is recommended in guidance for developing complex interventions, and can facilitate ownership and engagement with interventions [47]. Involving young leaders in the development of training, including but not limited to timings, may enhance the acceptability and credibility of training programmes, and should be tested in future research.

A consensus was reached for the statements that schools and community settings are appropriate settings to recruit young physical activity leaders. The existing literature largely focuses on school-based contexts, which may exclude older young people from participating. Additionally, rapid review findings indicate that community-based young leader training programmes may be more feasible and acceptable than school-based programmes, due to context-specific challenges such as restrictive timetables [42], and local community groups and organisations often have access to underserved groups who may stand to benefit most from young leadership training. Community-based physical activity young leader training programmes should be developed, delivered and evaluated to provide a more nuanced understanding about what works, for whom, and in what context.

Throughout the study, a distinction was made between characteristics that young leaders should possess prior to training (such as enthusiasm), and those that can be developed through training (such as adaptability to different circumstances). This has implications for the criteria used when recruiting young people to become physical activity leaders, but also highlights how training programmes can facilitate change and benefit the young people across a spectrum of skills and characteristics. This supports the concept that young leadership programmes have and should have a ‘dual benefit’, benefiting both the leaders and those they then deliver to. The Delphi consensus indicated that being physically active prior to training should not be a prerequisite, as there is an opportunity for more significant impact through recruiting inactive young people and supporting them to become active. However, there was a consensus around a range of traits, including confidence and responsibility, which young leaders should possess prior to training; this somewhat conflicts with a positive youth development perspective that all people have capacity to be leaders, and that investing in disadvantaged and undervalued young people can help address inequality [48]. Indeed, the value of supporting disengaged young people was articulated by various stakeholders as part of our qualitative consultations. The challenges and benefits of developing young people with and without pre-existing skills and characteristics should be explored in future research.

Despite not achieving consensus across the first two questionnaires, there were seven statements that the young leaders agreed should be included. This suggests that there may be a fundamental difference in the perspectives of young leaders compared to stakeholders involved in their recruitment and training. Opinion is mixed on whether ‘service users’ can be considered experts in Delphi methodology [49]. We believe that young leaders have valuable knowledge and opinions that may help challenge views of traditional ‘experts’. We therefore argue that the integration of young leader voices in this study and in wider young leader training programme development and evaluation increases the relevance, acceptability and potential impact of the work.

### Limitations

A limitation of this study is that only a small proportion of questionnaire respondents were young leaders. We experienced difficulty in accessing a high volume of young physical activity leaders through organizations, due to ethics/safeguarding protocols. Thus, we conducted an additional focus group with a small group of young leaders we were able to access, to ensure that young physical activity leaders were represented in this work.

The rapid review’s function was to inform the subsequent Delphi process. As a result, a rapid review method was chosen to ensure the relevant data was collated prior to the start of the Delphi work. Nevertheless, while the work was conducted systematically the overall process was less rigorous by nature. Only a small number of studies were identified in the rapid review (n = 5), and so the items generated from these studies, included in the current study, could be perceived as ungeneralizable. However, we argue that the review should be understood as a ‘starting point’ for the idea generation phase of the Delphi process, in conjunction with the initial focus groups and interviews, rather than a standalone ‘finding’. Additionally, the limited number of studies included in the review highlights the importance of taking an approach such as the Delphi method to develop a consensus on ‘what works’ to develop young physical activity leaders, to support the evidence-based development of training programmes in research and practice, given the relative dearth of evidence available within existing literature.

We did not collect demographic data such as gender and work experience from participants, therefore we do not know whether our sample is representative of the stakeholder groups. Additionally, the online, questionnaire-based nature of the study impacted participants’ capacity to express nuanced views, particularly those who were not involved in the consultation phase. In an attempt to mitigate this, the first questionnaire featured an open-ended question. This was an important addition, as 24 of the 32 new items arising from the open-ended question were integrated into the final toolkit. There were a number of experts contacted who were absent from work and unable to participate in the study, which limited the response rate. While other Delphi studies have reported higher ratios of responses to invites [50], the quantity of responses was comparatively higher in the current study, due to the volume of participants invited to participate. Furthermore, the response rate for questionnaires two and three were on par with or exceeded that of other similar studies. This is reflective of the interest in the topic, as well as the expanse of practitioner expertise and experience that has previously not been captured in research.

### Recommendations

The study findings were derived from the valuable, lived experience of those who work in the field of young leadership and thus strengthen the relationship between research and practice. This study provides recommendations for researchers and practitioners involved in designing and delivering young physical activity leader training programmes. For example, a blanket training package is unlikely to be appropriate; instead, programmes should offer a variety of training methods to suit different learning abilities and lifestyles. Content focused around physical activity, both theoretical and practical, should form a large component of the training, in addition to leadership-based skill-building content. It may also be beneficial for training facilitators to consider implementing more community-based training as opposed to using schools; this appears to facilitate young leader diversity, as well as avoiding some of the institutional challenges that come with using academic settings to recruit young leaders. We are currently applying learning from this study to inform the development and evaluation of JU:MP Leads, as part of the Bradford Local Delivery Pilot (https://www.activebradford.com/ju-mp-leads-programme).

## Conclusion

This study generated a consensus on important considerations for developing effective young physical activity leader training programmes that can be used to inform future research, policy and academic practice. Co-produced, community-based training methods for young leaders may be the most appropriate and effective for creating behavior change. The lack of research in this area highlights a need to both develop new, and identify existing community-based training programmes, to further understand what works, how, for whom and under what conditions, when identifying and training young people to become physical activity leaders.

## Supporting information

S1 Table MMAT results. (DOCX)

S1 File Delphi survey round 1. (DOCX)

S2 File Delphi survey round 2. (DOCX)

S3 File Delphi survey round 3. (DOCX)
