# Supplementary material for: How to develop young physical activity leaders? A Delphi study
Source: PLoS One. 2023 Sep 29;18(9):e0286920. doi: 10.1371/journal.pone.0286920 (PMC10540972; doi:10.1371/journal.pone.0286920)
Supplement: S1 Table — (DOCX) [file pone.0286920.s001.docx]

Appendix 1 - MMAT results


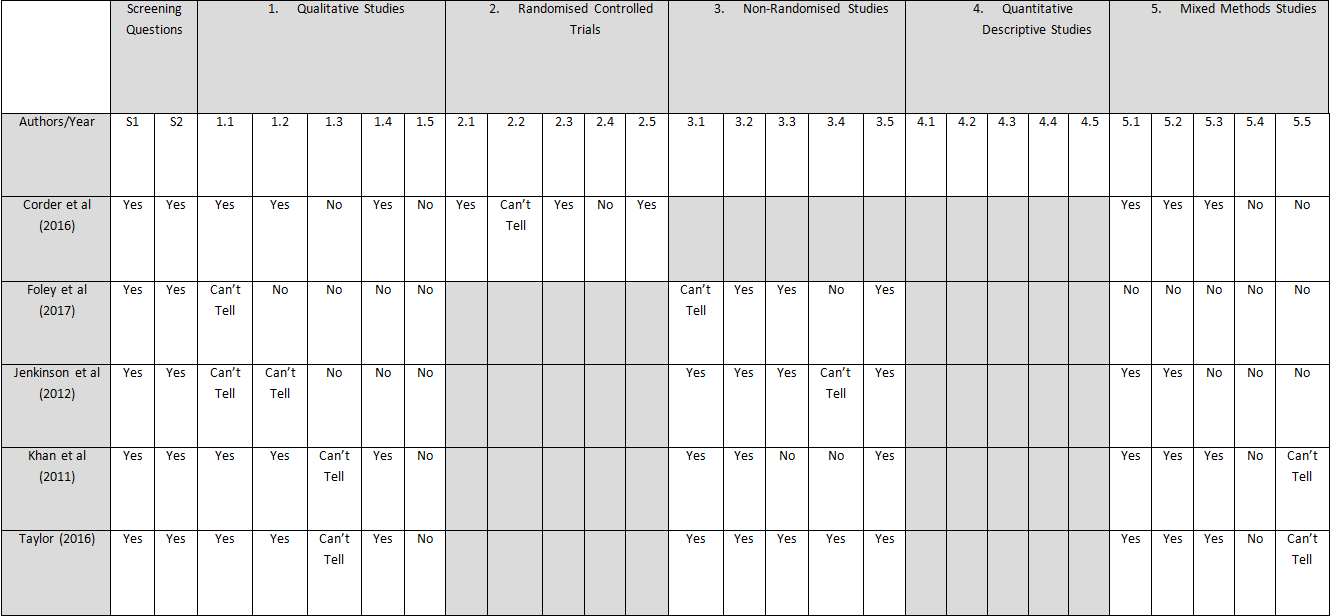


S1. Are there clear research questions? S2. Do the collected data allow to address the research questions? 1.1. Is the qualitative approach appropriate to answer the research question? 1.2. Are the qualitative data collection methods adequate to address the research question? 1.3. Are the findings adequately derived from the data? 1.4. Is the interpretation of results sufficiently substantiated by data? 1.5. Is there coherence between qualitative data sources, collection, analysis and interpretation? 2.1. Is randomization appropriately performed? 2.2. Are the groups comparable at baseline? 2.3. Are there complete outcome data? 2.3. Are there complete outcome data? 2.4. Are outcome assessors blinded to the intervention provided? 2.5 Did the participants adhere to the assigned intervention? 3.1 Are the participants representative of the target population? 3.2 Are measurements appropriate regarding both the outcome and intervention? 3.3 Are there complete outcome data? 3.4 Are the confounders accounted for in the design and analysis? 3.5 During the study period, is the intervention administered (or exposure occurred) as intended? 4.1. Is the sampling strategy relevant to address the research question? 4.2. Is the sample representative of the target population? 4.3. Are the measurements appropriate? 4.4. Is the risk of nonresponse bias low? 4.5. Is the statistical analysis appropriate to answer the research question? 5.1. Is there an adequate rationale for using a mixed methods design to address the research question? 5.2. Are the different components of the study effectively integrated to answer the research question? 5.3. Are the outputs of the integration of qualitative and quantitative components adequately interpreted? 5.4. Are divergences and inconsistencies between quantitative and qualitative results adequately addressed? 5.5. Do the different components of the study adhere to the quality criteria of each tradition of the methods involved?
