# Supplementary material for: How to develop young physical activity leaders? A Delphi study
Source: PLoS One. 2023 Sep 29;18(9):e0286920. doi: 10.1371/journal.pone.0286920 (PMC10540972; doi:10.1371/journal.pone.0286920)
Supplement: S1 File — (DOCX) [file pone.0286920.s002.docx]

Appendix 2 - delphi survey round 1

**1. Please select: ***

| 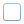 | I confirm that I have read the information above and I consent to taking part. |
| --- | --- |
| 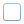 | I do not consent to taking part in the study |

The statements included in this questionnaire are based upon **consultations** the team has done with various stakeholders who work within the field, and also from a **review of relevant literature** on the topic.

**You will have a chance at the end of this questionnaire to add anything that you feel is missing from the questionnaire.**

All of the statements included will be in relation to developing a **successful young physical activity leader**. When we talk about a successful young physical activity leader, we are thinking about someone aged 16-25 who successfully supports children and young people to engage in physical activity.

We would like to know your opinion; **there are no right or wrong answers**.

1. **Before training: characteristics and traits a young person should have**

This page will have a number of statements surrounding the **characteristics and traits** that a young person should have, prior to their training. These are things that you think, ideally, **a young person should have before** taking them on to train as a young physical activity leader.

**We would like you to indicate your level of agreement with each statement, with 1 being "Strongly Disagree" and 9 being "Strongly Agree"**

###

### **"It is essential for a young person to ________________, before becoming a young physical activity leader"**

|  | 1- Strongly disagree | 2 | 3 | 4 | 5 | 6 | 7 | 8 | 9- Strongly agree |
| --- | --- | --- | --- | --- | --- | --- | --- | --- | --- |
| have **grown up** in the area in which they are delivering physical activity | 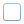 | 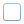 | 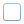 | 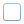 | 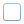 | 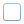 | 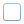 | 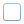 | 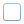 |
| **live** in the area that they will deliver physical activity in | 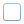 | 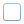 | 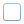 | 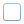 | 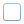 | 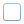 | 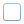 | 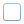 | 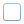 |
| **look like** and be **representative** of the the individuals who receive the physical activity | 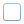 | 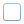 | 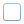 | 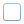 | 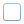 | 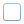 | 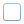 | 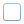 | 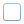 |
| **appear confident** (e.g., being confident in talking to the community, trainers and peers) | 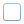 | 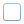 | 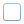 | 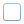 | 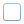 | 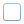 | 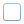 | 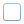 | 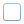 |
| have **belief** in their own **leadership abilities** | 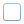 | 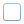 | 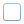 | 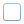 | 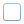 | 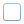 | 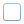 | 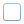 | 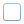 |
| be **enthusiastic** | 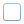 | 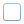 | 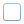 | 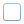 | 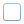 | 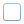 | 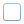 | 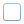 | 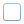 |
| have a **willingness** to be involved | 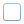 | 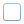 | 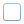 | 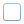 | 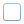 | 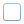 | 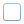 | 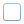 | 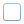 |
| have a **passion** for physical activity | 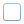 | 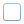 | 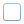 | 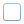 | 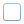 | 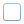 | 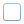 | 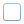 | 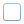 |
| be **physically active themselves** | 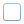 | 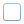 | 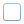 | 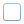 | 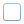 | 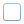 | 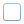 | 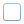 | 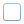 |
| have a **positive mindset** | 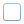 | 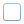 | 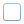 | 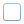 | 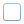 | 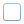 | 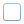 | 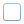 | 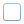 |
| be **trustworthy** | 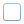 | 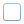 | 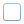 | 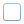 | 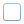 | 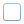 | 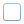 | 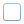 |  |
| be **disciplined** |  |  |  |  |  |  |  |  |  |
| be **responsible** |  |  |  |  |  |  |  |  |  |
| be **honest** |  |  |  |  |  |  |  |  |  |
| be **patient** |  |  |  |  |  |  |  |  |  |
| be **punctual** |  |  |  |  |  |  |  |  |  |
| have a **good standard of English** |  |  |  |  |  |  |  |  |  |

1. **Before training- recruitment of young physical activity leaders**

This page will have a number of statements surrounding the **recruitment** of young physical activity leaders.

**" _____________ are an effective place to identify, attract and recruit good-quality young physical activity leader candidates from."**

|  | 1-Strongly disagree | 2 | 3 | 4 | 5 | 6 | 7 | 8 | 9- Strongly agree |
| --- | --- | --- | --- | --- | --- | --- | --- | --- | --- |
| School settings |  |  |  |  |  |  |  |  |  |
| Religious settings |  |  |  |  |  |  |  |  |  |
| Social media platforms |  |  |  |  |  |  |  |  |  |
| Community organisations |  |  |  |  |  |  |  |  |  |

1. **Training: Content**

This page will have a number of statements surrounding the **content** of young physical activity leaders' training.

**We would like you to indicate your level of agreement with each statement.**

###

### **"________________ should be embedded within training for young physical activity leaders".**

|  | 1- Strongly disagree | 2 | 3 | 4 | 5 | 6 | 7 | 8 | 9- Strongly agree |
| --- | --- | --- | --- | --- | --- | --- | --- | --- | --- |
| Learning to **session plan** |  |  |  |  |  |  |  |  |  |
| Learning to **carry out risk-assessments** |  |  |  |  |  |  |  |  |  |
| Learning **first aid** |  |  |  |  |  |  |  |  |  |
| Learning about **equality and diversity within sport and society** |  |  |  |  |  |  |  |  |  |
| Learning to **goal-set** |  |  |  |  |  |  |  |  |  |
| Learning about **safeguarding** |  |  |  |  |  |  |  |  |  |
| Learning **basic skills in languages that are relevant to the community** (e.g., Punjabi, Urdu, British Sign Language etc.) |  |  |  |  |  |  |  |  |  |
| Learning about the **history of the community** they are delivering in |  |  |  |  |  |  |  |  |  |
| Learning about the **barriers to physical activity within the community** they are delivering in |  |  |  |  |  |  |  |  |  |
| Learning about **different sports and physical activities** |  |  |  |  |  |  |  |  |  |
| Learning about **the importance of being physically active** |  |  |  |  |  |  |  |  |  |

1. **Training: Format and Context**

This page will have a number of statements surrounding the **format** and **context** *(e.g., setting, timing)* of young physical activity leaders' training.

**We would like you to indicate your level of agreement with each statement, with 1 being "Strongly Disagree" and 9 being "Strongly Agree"**

###

### **"Training courses for young physical activity leaders should..."**

|  | 1- Strongly Disagree | 2 | 3 | 4 | 5 | 6 | 7 | 8 | 9- Strongly Agree |
| --- | --- | --- | --- | --- | --- | --- | --- | --- | --- |
| **offer flexible timings for training,** that fit each individual leader's availability. |  |  |  |  |  |  |  |  |  |
| be delivered in a **'little and often'** way, over **longer time periods** (e.g., for one hour a week over six months) |  |  |  |  |  |  |  |  |  |
| be a **structured programme** with **pre-defined elements** |  |  |  |  |  |  |  |  |  |
| be delivered in **larger chunks** to be completed **more quickly** (e.g., over one or two full-days). |  |  |  |  |  |  |  |  |  |
| be delivered **in-person** |  |  |  |  |  |  |  |  |  |
| be delivered **online** |  |  |  |  |  |  |  |  |  |
| be delivered via a **blend** of **online and in-person** |  |  |  |  |  |  |  |  |  |
| be delivered via **formal styles of learning (such as presentations, lessons or workbooks)** |  |  |  |  |  |  |  |  |  |
| be delivered via **informal, less structured methods** |  |  |  |  |  |  |  |  |  |
| give young physical activity leaders the opportunity to **tailor the content** of the training to **match their interests** |  |  |  |  |  |  |  |  |  |
| give young physical activity leaders the opportunity to **shadow experienced leaders** |  |  |  |  |  |  |  |  |  |
| give young physical activity leaders the opportunity to **practice leading sessions** |  |  |  |  |  |  |  |  |  |
| give young physical activity leaders the opportunity to **undertake group work** |  |  |  |  |  |  |  |  |  |
| give young physical activity leaders the opportunity to **reflect on their training 'journey'** |  |  |  |  |  |  |  |  |  |
| teach young physical activity leaders how to **engage in reflective practice** |  |  |  |  |  |  |  |  |  |
| be delivered by **other young physical activity leaders** |  |  |  |  |  |  |  |  |  |
| be delivered by **individuals from local organisations** (who could be considered experts in a certain sport) |  |  |  |  |  |  |  |  |  |
| be delivered by **the youth service** |  |  |  |  |  |  |  |  |  |
| be delivered by **schools** |  |  |  |  |  |  |  |  |  |

1. **Training: Incentives and Rewards**

This page will have a number of statements surrounding the use of **incentives and rewards** as part of young physical activity leaders' training.

**We would like you to indicate your level of agreement with each statement, with 1 being "Strongly Disagree" and 9 being "Strongly Agree"**

|  | 1- Strongly Disagree | 2 | 3 | 4 | 5 | 6 | 7 | 8 | 9- Strongly Agree |
| --- | --- | --- | --- | --- | --- | --- | --- | --- | --- |
| It is important that young physical activity leaders are **paid** for any sessions they lead as part of their training |  |  |  |  |  |  |  |  |  |
| It is important that young physical activity leader's training includes **formal qualifications** |  |  |  |  |  |  |  |  |  |
| It is important that young physical activity leader's **travel expenses to and from training** are covered |  |  |  |  |  |  |  |  |  |
| It is important that young physical activity leader's **food and drink is provided** as part of their training |  |  |  |  |  |  |  |  |  |
| It is important that the cost of **equipment used as part of training** is covered |  |  |  |  |  |  |  |  |  |
| **No external incentives** are required to be included in young physical activity leader's training. |  |  |  |  |  |  |  |  |  |

1. **Outcomes of Training- Relationship building**

This page will have a number of statements surrounding the **relationships** that young physical activity leaders with others around them.

**We would like you to indicate your level of agreement with each statement, with 1 being "Strongly Disagree" and 9 being "Strongly Agree"**

### **"It is important that young physical activity leaders develop good relationships with ________________ during their training"**

|  | 1- Strongly Disagree | 2 | 3 | 4 | 5 | 6 | 7 | 8 | 9-Strongly Agree |
| --- | --- | --- | --- | --- | --- | --- | --- | --- | --- |
| other young leaders |  |  |  |  |  |  |  |  |  |
| their trainers |  |  |  |  |  |  |  |  |  |
| local organisations |  |  |  |  |  |  |  |  |  |
| members of the community |  |  |  |  |  |  |  |  |  |

1. **Outcomes of Training: Skills Development**

This page will have a number of statements surrounding the **skills** that might need to be developed throughout young physical activity leaders' training.

When we talk about a **successful young physical activity leader**, we are thinking about someone aged 16-25 who successfully supports children and young people to increase their physical activity levels.

**We would like you to indicate your level of agreement with each statement, with 1 being "Strongly Disagree" and 9 being "Strongly Agree"**

###

### **"It is essential for a young physical activity leader to develop their _________________ during their training"**

|  | 1-Strongly disagree | 2 | 3 | 4 | 5 | 6 | 7 | 8 | 9- Strongly agree |
| --- | --- | --- | --- | --- | --- | --- | --- | --- | --- |
| **listening** skills |  |  |  |  |  |  |  |  |  |
| ability to **motivate others** |  |  |  |  |  |  |  |  |  |
| **communication** skills |  |  |  |  |  |  |  |  |  |
| **presentation** skills |  |  |  |  |  |  |  |  |  |
| **initiative** |  |  |  |  |  |  |  |  |  |
| **team-working** skills |  |  |  |  |  |  |  |  |  |
| ability to **use social media effectively** |  |  |  |  |  |  |  |  |  |

1. **Characteristics and Traits (POST-training)**

This page will have a number of statements surrounding the **characteristics and traits** that a young physical activity leader should have **once they have completed their training.**

**We would like you to indicate your level of agreement with each statement, with 1 being "Strongly Disagree" and 9 being "Strongly Agree”**

###

### **"It is essential for a young physical activity leader to ____________________ once they have completed their training"**

|  | 1- Strongly disagree | 2 | 3 | 4 | 5 | 6 | 7 | 8 | 9- Strongly agree |
| --- | --- | --- | --- | --- | --- | --- | --- | --- | --- |
| **appear confident** (e.g., being confident in talking to the community, trainers and peers) |  |  |  |  |  |  |  |  |  |
| **have belief** in their own **leadership abilities** |  |  |  |  |  |  |  |  |  |
| be **enthusiastic** |  |  |  |  |  |  |  |  |  |
| have a **willingness** to be involved |  |  |  |  |  |  |  |  |  |
| have a **passion** for physical activity |  |  |  |  |  |  |  |  |  |
| be **physically active themselves** |  |  |  |  |  |  |  |  |  |
| have a **positive mindset** |  |  |  |  |  |  |  |  |  |
| be **trustworthy** |  |  |  |  |  |  |  |  |  |
| be **disciplined** |  |  |  |  |  |  |  |  |  |
| be **responsible** |  |  |  |  |  |  |  |  |  |
| be **honest** |  |  |  |  |  |  |  |  |  |
| be **patient** |  |  |  |  |  |  |  |  |  |
| be **punctual** |  |  |  |  |  |  |  |  |  |
| have a **good standard of English** |  |  |  |  |  |  |  |  |  |

1. **About you**

**Do you work in any of the roles/sectors below? (Please select all that apply):**

|  | Voluntary |
| --- | --- |
|  | Council- not including youth service |
|  | Youth Service |
|  | JU:MP employee |
|  | Activity Provider |
|  | Religious Setting |

|  | School teacher or employee |
| --- | --- |
|  | Academia/Research |
|  | Young physical activity leader |
|  | Student |
|  | Other (please specify): |

**Are you aged 25 or under?**

|  | Yes, I am 25 years of age or younger |
| --- | --- |
|  | No, I am over 25 years of age |

### **Is there anything that isn’t covered in this questionnaire, that you think is important to include? This might be either in terms of the characteristics young physical activity leaders should have, and the training that they receive.**
