# Supplementary material for: How to develop young physical activity leaders? A Delphi study
Source: PLoS One. 2023 Sep 29;18(9):e0286920. doi: 10.1371/journal.pone.0286920 (PMC10540972; doi:10.1371/journal.pone.0286920)
Supplement: S2 File — (DOCX) [file pone.0286920.s003.docx]

Appendix 3 - delphi survey round 2

**Please select: ***

| 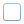 | I confirm that I have read the information above and I agree to taking part. |
| --- | --- |
| 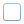 | I do not agree to take part in the study. |

**Part 1: Statements that reached consensus from round 1**

Below are the 49 items from the first questionnaire round that did reach a consensus. This means that over 75% of the participants in the study agreed with the statement (scores of **7**, **8** or **9**). You will not be scoring these items again.

[list of statements]

**Part 2: Open to reisions from previous questionnaire**

This section contains statements that were previously seen in the first questionnaire round. These statements did not reach the threshold for consensus from the overall sample, and therefore have been inputted into this second questionnaire to give you the opportunity to revise your answers if you wish.

You should have received a table, along with the email that contained the link for this questionnaire. On that table, it summarizes the answer that **you** gave in round 1, the average score your **stakeholder group** gave (*We have used answers from round 1, and have categorized each participant into the 'best-fit', broader role/sector category; please do let us know via email if you feel as though this category is incorrect*), and the average score that the **whole sample** gave, for each item that hasn't reached the threshold for consensus.

**Please use the table in the email to help you reflect on the scores you gave each item in round 1.**

If you do not wish to revise your answer upon reflection, please just select the **same score that you gave in round 1**.

Please rate how much you agree or disagree to the statements below from **1 (strongly disagree)** to **9 (strongly agree)**:

**Having had the opportunity to reflect on your answers, the average of your stakeholder group and the answers of the overall group, please rate how much you agree or disagree with the following statements, from 1 (strongly disagree) to 9 (strongly agree):**

### **"It is essential for a young person to ________________, before becoming a young physical activity leader"**

|  | 1 - Strongly Disagree | 2 | 3 | 4 | 5 | 6 | 7 | 8 | 9 - Strongly Agree |
| --- | --- | --- | --- | --- | --- | --- | --- | --- | --- |
| **live** in the area they will deliver physical activity in | 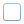 | 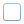 | 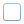 | 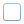 | 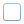 | 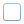 | 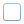 | 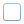 | 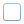 |
| have **grown up in the area** in which they are delivering physical activity | 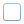 | 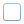 | 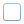 | 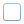 | 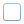 | 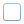 | 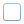 | 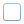 | 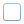 |
| **look like** and be **representative** of the individuals who receive the physical activity | 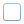 | 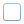 | 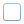 | 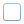 | 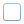 | 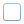 | 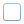 | 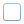 | 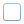 |
| appear **confident** (e.g., being confident in talking to the community, trainers and peers) | 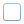 | 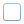 | 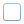 | 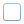 | 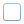 | 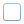 | 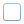 | 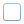 | 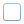 |
| have **belief in their own leadership abilities** | 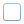 | 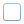 | 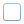 | 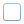 | 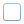 | 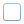 | 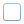 | 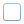 | 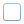 |
| be **physically active themselves** | 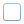 | 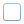 | 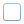 | 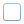 | 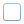 | 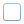 | 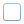 | 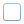 | 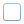 |
| have a good **standard of English** | 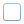 | 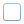 | 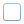 | 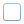 | 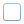 | 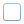 | 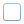 | 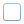 | 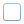 |

### **3.**

### **" _____________ are an effective place to identify, attract and recruit good-quality young physical activity leader candidates from.**

|  | 1 - Strongly Disagree | 2 | 3 | 4 | 5 | 6 | 7 | 8 | 9 - Strongly Agree |
| --- | --- | --- | --- | --- | --- | --- | --- | --- | --- |
| **Religious Settings** | 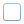 | 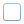 | 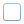 | 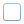 | 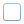 | 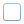 | 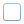 | 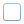 | 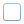 |
| **Social Media Platforms** | 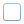 | 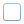 | 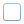 | 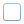 | 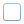 | 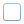 | 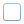 | 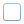 | 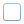 |

### **"________________ should be embedded within training for young physical activity leaders".**

|  | 1 - Strongly Disagree | 2 | 3 | 4 | 5 | 6 | 7 | 8 | 9 - Strongly Agree |
| --- | --- | --- | --- | --- | --- | --- | --- | --- | --- |
| Learning about the **history of the community** they are delivering in | 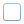 | 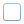 | 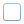 | 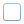 | 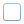 | 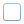 | 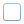 | 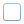 | 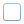 |
| Learning to **goal-set** | 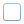 | 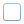 | 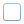 | 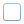 | 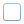 | 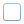 | 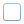 | 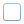 |  |
| Learning **basic skills in languages** that are relevant to the community (e.g., Punjabi, Urdu, British Sign Language etc.) |  |  |  |  |  |  |  |  |  |
| Learning about **different sports and physical activities** |  |  |  |  |  |  |  |  |  |

### **"Training courses for young physical activity leaders should..."**

|  | 1 - Strongly Disagree | 2 | 3 | 4 | 5 | 6 | 7 | 8 | 9 - Strongly Agree |
| --- | --- | --- | --- | --- | --- | --- | --- | --- | --- |
| be delivered in a **'little and often'** way, over **longer time periods** (e.g., for one hour a week over six months) |  |  |  |  |  |  |  |  |  |
| be delivered in **larger chunks** to be completed more quickly (e.g., over one or two full-days). |  |  |  |  |  |  |  |  |  |
| offer **flexible timings for training** that fit with each individual leader's availability |  |  |  |  |  |  |  |  |  |
| be a **structured programme** with pre-defined elements |  |  |  |  |  |  |  |  |  |
| be delivered **online** |  |  |  |  |  |  |  |  |  |
| be delivered via **formal styles of learning** (such as presentations, lessons or workbooks) |  |  |  |  |  |  |  |  |  |
| be delivered via a **blend of online and in-person** |  |  |  |  |  |  |  |  |  |
| be delivered via **informal, less structured methods** |  |  |  |  |  |  |  |  |  |
| give young physical activity leaders the **opportunity to tailor the content** of the training to match their interests |  |  |  |  |  |  |  |  |  |
| be delivered by the **Youth Service** |  |  |  |  |  |  |  |  |  |
| be delivered by **schools** |  |  |  |  |  |  |  |  |  |
| be delivered by **other young physical activity leaders** |  |  |  |  |  |  |  |  |  |
| be delivered by **individuals from local organisations** (who could be considered experts in a certain sport) |  |  |  |  |  |  |  |  |  |

**Please rate how much you agree or disagree with the following statements, from 1 (Strongly Disagree) to 9 (Strongly Agree)**

|  | 1 - Strongly Disagree | 2 | 3 | 4 | 5 | 6 | 7 | 8 | 9 - Strongly Agree |
| --- | --- | --- | --- | --- | --- | --- | --- | --- | --- |
| It is important that young physical activity leaders are **paid** for any sessions they lead as part of their training |  |  |  |  |  |  |  |  |  |
| It is important that young physical activity leader's training includes **formal qualifications** |  |  |  |  |  |  |  |  |  |
| It is important that young physical activity leader's **food and drink is provided** as part of the training |  |  |  |  |  |  |  |  |  |
| **No external incentives** are required to be included in young physical activity leader's training |  |  |  |  |  |  |  |  |  |

### **"It is essential for a young physical activity leader to develop their _________________ during their training"**

|  | 1 - Strongly Disagree | 2 | 3 | 4 | 5 | 6 | 7 | 8 | 9 - Strongly Agree |
| --- | --- | --- | --- | --- | --- | --- | --- | --- | --- |
| an ability to **use social media effectively** |  |  |  |  |  |  |  |  |  |

### **"It is essential for a young physical activity leader to ____________________ once they have completed their training"**

|  | 1 - Strongly Disagree | 2 | 3 | 4 | 5 | 6 | 7 | 8 | 9 - Strongly Agree |
| --- | --- | --- | --- | --- | --- | --- | --- | --- | --- |
| be **physically active themselves** |  |  |  |  |  |  |  |  |  |
| have a **good standard of English** |  |  |  |  |  |  |  |  |  |

**Part 3: New items from previous questionnaire**

This section of the questionnaire contains **new** items that were generated from the open-text box that was at the end of the first questionnaire round. Some of these items are completely new, while others are items from round 1 that have been edited for clarity. As these are newly-generated, there are no previous scores to reflect on. We are simply asking for your own, personal score of agreement on these items.

Please rate your level of agreement from 1 **(strongly disagree)** to **9 (strongly agree)**:

**"It is essential for a young person to be ________________, before becoming a young physical activity leader"**

|  | 1 - Strongly Disagree | 2 | 3 | 4 | 5 | 6 | 7 | 8 | 9 - Strongly Agree |
| --- | --- | --- | --- | --- | --- | --- | --- | --- | --- |
| **empathetic** |  |  |  |  |  |  |  |  |  |
| **resilient** |  |  |  |  |  |  |  |  |  |
| **creative** |  |  |  |  |  |  |  |  |  |
| **actively involved** in the community/voluntary sector |  |  |  |  |  |  |  |  |  |
| willing to **learn** new skills |  |  |  |  |  |  |  |  |  |
| comfortable with a **role of responsibility** |  |  |  |  |  |  |  |  |  |

**"________________ should be embedded within training for young physical activity leaders".**

|  | 1 - Strongly Disagree | 2 | 3 | 4 | 5 | 6 | 7 | 8 | 9 - Strongly Agree |
| --- | --- | --- | --- | --- | --- | --- | --- | --- | --- |
| **CV development and support** |  |  |  |  |  |  |  |  |  |
| Learning how to **deliver sport and physical activity** |  |  |  |  |  |  |  |  |  |
| Learning about the **history and rules of specific sports** |  |  |  |  |  |  |  |  |  |
| Learning about the **benefits and importance of physical activity** (e.g. the link between physical activity and mental health) |  |  |  |  |  |  |  |  |  |

**"Training packages for young physical activity leaders should..."**

|  | 1 - Strongly Disagree | 2 | 3 | 4 | 5 | 6 | 7 | 8 | 9 - Strongly Agree |
| --- | --- | --- | --- | --- | --- | --- | --- | --- | --- |
| be delivered in a **mixture of settings** (e.g. some inside and some outside) |  |  |  |  |  |  |  |  |  |
| provide a **mentor** for the young leader to learn from |  |  |  |  |  |  |  |  |  |
| give the young leaders **real world practice** through securing **placement** opportunities |  |  |  |  |  |  |  |  |  |
| encourage all young leaders to get involved **equally** |  |  |  |  |  |  |  |  |  |
| help secure **work opportunities** for the young leaders following the training |  |  |  |  |  |  |  |  |  |
| include a **supplementary guide or document** to support young leaders in their current training |  |  |  |  |  |  |  |  |  |
| include a **supplementary guide or document** for young leaders to refer to once they've completed training |  |  |  |  |  |  |  |  |  |

**"It is essential for a young physical activity leader to develop their _________________ during their training"**

|  | 1 - Strongly Disagree | 2 | 3 | 4 | 5 | 6 | 7 | 8 | 9 - Strongly Agree |
| --- | --- | --- | --- | --- | --- | --- | --- | --- | --- |
| **organisational** skills |  |  |  |  |  |  |  |  |  |
| **problem solving** skills |  |  |  |  |  |  |  |  |  |
| **specific movement skills** and how to apply them to sport and physical activity |  |  |  |  |  |  |  |  |  |
| **interpersonal** skills |  |  |  |  |  |  |  |  |  |
| **behaviour management** skills |  |  |  |  |  |  |  |  |  |
| the ability to **generate positive emotions** within a group |  |  |  |  |  |  |  |  |  |
| the ability to **have fun** while leading sessions |  |  |  |  |  |  |  |  |  |

### **13. "It is essential for a young physical activity leader to ____________________ once they have completed their training"**

|  | 1 - Strongly Disagree | 2 | 3 | 4 | 5 | 6 | 7 | 8 | 9 - Strongly Agree |
| --- | --- | --- | --- | --- | --- | --- | --- | --- | --- |
| be able to **adapt to different circumstances** |  |  |  |  |  |  |  |  |  |
| ensure sessions are **inclusive** for all involved |  |  |  |  |  |  |  |  |  |
| be able to experiment with **different approaches to sessions** |  |  |  |  |  |  |  |  |  |
| be **empathetic** |  |  |  |  |  |  |  |  |  |
| be **resilient** |  |  |  |  |  |  |  |  |  |
| be **creative** |  |  |  |  |  |  |  |  |  |
| be **actively involved** in the community/voluntary sector |  |  |  |  |  |  |  |  |  |
| be comfortable with a **role of responsibility** |  |  |  |  |  |  |  |  |  |
