# Supplementary material for: How to develop young physical activity leaders? A Delphi study
Source: PLoS One. 2023 Sep 29;18(9):e0286920. doi: 10.1371/journal.pone.0286920 (PMC10540972; doi:10.1371/journal.pone.0286920)
Supplement: S3 File — (DOCX) [file pone.0286920.s004.docx]

Appendix 4 - delphi survey round 3

**Part 1:** Below are the items from the previous 2 questionnaire rounds that did reach a consensus. This means that over 75% of the participants in the study agreed with the statement (scores of 7, 8 or 9). You will not be scoring these items again. [listed items]

**Part 2: Near-consensus items that received feedback in questionnaire rounds 1 AND 2**

The following items have been rated twice- once in the first questionnaire you completed, and also the second (as they didn't reach consensus in the first questionnaire). They have been included as, while they didn't reach a 75% agreement even after the second questionnaire, they are considered **near-consensus** items (i.e., they had an agreement percentage between 65-75%). Because they were near-consensus, they were taken to the focus group with young leaders, who gave their opinions and feelings on them.

### **"It is essential for a young person to be physically active, before taking them on as a young physical activity leader"**

### The young leaders both agreed and disagreed with this statement. Some agreed this was essential, as it was important for a young leader to be a role model and it was felt that it was a key motivation to becoming a young physical activity leader. Others felt 'essential' was too strong of a word, but 'desirable' could be more accurate. There may even be benefit to not being physically active, as this could make the leader more open to different types of activities.

### **When considering the above, to what extent do you agree or disagree with the statement?**

| 1- Strongly disagree | 2 | 3 | 4 | 5 | 6 | 7 | 8 | 9- Strongly agree |
| --- | --- | --- | --- | --- | --- | --- | --- | --- |
| 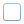 | 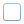 | 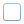 | 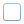 | 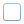 | 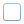 | 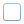 | 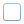 | 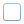 |

**"Training courses for young physical activity leaders should offer flexible timings for training that fit with each individual leader's availability"**

The young leaders mostly agreed with this statement, as it was felt it was important to fit within different individual's commitments and circumstances (e.g., childcare commitments), however it was also discussed that some set timings may be useful to build in structure to the training.

**When considering the above, to what extent do you agree or disagree with the statement?**

| 1- Strongly disagree | 2 | 3 | 4 | 5 | 6 | 7 | 8 | 9- Strongly agree |
| --- | --- | --- | --- | --- | --- | --- | --- | --- |
| 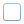 | 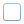 | 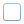 | 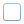 | 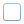 | 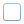 | 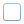 | 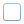 | 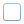 |

**"Training courses for young physical activity leaders should be delivered by individuals from local organisations"**

The young leaders agreed with this statement, however it was contested if being local was important. From their experience, where a different range of organisations did deliver certain training elements, they felt positively about it- saying it also gave them opportunities to network and build connections and relationships, as well as learning from experts in the activity.

**When considering the above, to what extent do you agree or disagree with the statement?**

| 1- Strongly disagree | 2 | 3 | 4 | 5 | 6 | 7 | 8 | 9- Strongly agree |
| --- | --- | --- | --- | --- | --- | --- | --- | --- |
| 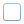 | 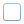 | 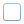 | 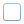 | 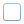 | 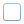 | 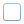 | 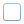 | 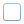 |

**"Training courses for young physical activity leaders should offer formal qualifications"**

The young leaders agreed with this statement. They felt it was important as it a) would be a motivator for getting young people on the training course, and b) set young people up for future employment. The young leaders indicated that they themselves would not have joined their course if it did not offer formal qualifications.

**When considering the above, to what extent do you agree or disagree with the statement?**

| 1- Strongly disagree | 2 | 3 | 4 | 5 | 6 | 7 | 8 | 9- Strongly agree |
| --- | --- | --- | --- | --- | --- | --- | --- | --- |
| 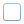 | 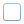 | 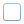 | 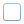 | 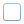 | 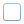 | 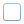 | 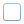 | 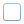 |

**"It is essential for a young physical activity leader to be physically active themselves once they have completed their training"**

The young leaders agreed with this statement. They felt that it is important for young leaders to role model. physical activity. It was also felt that being physically active would make you a better leader, for example through experience of taking part in certain sports, or through increased fitness.

**When considering the above, to what extent do you agree or disagree with the statement?**

| 1- Strongly disagree | 2 | 3 | 4 | 5 | 6 | 7 | 8 | 9- Strongly agree |
| --- | --- | --- | --- | --- | --- | --- | --- | --- |
| 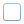 | 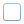 | 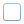 | 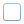 | 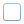 | 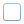 | 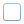 | 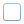 | 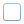 |

**Part 3: Near-consensus items that received feedback in questionnaire round 2 ONLY**

The following items are items which were only rated once, in the second (as they were generated from the free-text box at the end of questionnaire 1). They have been included as, because they are **near-consensus** items (i.e., they had an agreement percentage between 65-75%), they were taken to the focus group with young leaders.

**"It is essential for a young person to be empathetic, before taking them on as a young physical activity leader"**

The young leaders agreed with this statement. This was because it was felt that, while empathy was fundamental, it would be difficult to develop in such a short period of time (i.e., over the course of a training period)

**When considering the above, to what extent do you agree or disagree with the statement?**

| 1- Strongly disagree | 2 | 3 | 4 | 5 | 6 | 7 | 8 | 9- Strongly agree |
| --- | --- | --- | --- | --- | --- | --- | --- | --- |
| 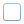 | 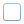 | 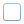 | 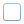 | 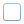 | 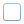 | 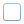 | 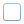 | 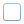 |

**"It is essential for a young person to be resilient, before taking them on as a young physical activity leader"**

The young leaders disagreed with this statement. It was agreed that resilience was an essential trait, however it was felt that this would be developed in training, through experiencing sessions for themselves- therefore not being an essential pre-requisite to becoming a young physical activity leader.

**When considering the above, to what extent do you agree or disagree with the statement?**

| 1- Strongly disagree | 2 | 3 | 4 | 5 | 6 | 7 | 8 | 9- Strongly agree |
| --- | --- | --- | --- | --- | --- | --- | --- | --- |
| 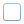 | 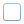 | 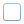 | 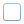 | 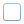 | 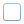 | 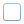 | 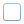 | 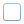 |

### **"It is essential for a young physical activity leader to have developed their specific movement skills and how to apply them to sport and physical activity, once they have completed their training"**

The young leaders mostly agreed with this statement. The importance of learning specific skills in relation to the prevention of injury was discussed, as well ensuring that children are shown these skills correctly when being taught. They felt like they might look 'silly' leading sessions if they didn't have these skills, and they also felt it may also impact the leader's ability to get qualifications or gain the trust of the children and young people they would deliver to. However, they felt like it wasn't necessary to learn the specifics of all sports, but just those a young leader would want to lead in the future (where learning specific skills is relevant- i.e., not for activities such as walking).

### **When considering the above, to what extent do you agree or disagree with the statement?**

| 1- Strongly disagree | 2 | 3 | 4 | 5 | 6 | 7 | 8 | 9- Strongly agree |
| --- | --- | --- | --- | --- | --- | --- | --- | --- |
| 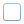 | 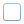 | 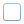 | 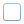 | 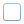 | 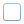 | 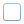 | 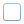 | 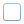 |

### **"It is essential for a young physical activity leader to be actively involved in the community/voluntary sector once they have completed their training"**

### The young leaders agreed with this statement, depending on if there were opportunities to become involved and the role one wanted to take. The major benefit of being involved was that you were visible and actively known within the community A benefit was that you were actively known in the community. They felt it was important for children to do things outside school and the community is such as important part of the job, so being a role model and visible within the community was essential.

###

### **When considering the above, to what extent do you agree or disagree with the statement?**

| 1- Strongly disagree | 2 | 3 | 4 | 5 | 6 | 7 | 8 | 9- Strongly agree |
| --- | --- | --- | --- | --- | --- | --- | --- | --- |
| 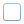 | 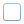 | 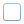 | 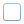 | 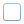 | 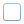 | 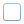 | 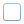 | 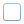 |

**Part 4: Non-consensus items that received feedback in questionnaire round 2 ONLY**

The following items were only rated once, in the second questionnaire (as they were generated from the free-text box at the end of questionnaire 1). These items were **not** taken to the focus group with young leaders, as they were **not** considered near-consensus, but they are included in the present questionnaire because they require a second round of feedback.

You can use the stakeholder average scores (found in your personalised feedback table) to help you reflect.

**"It is essential for a young person to be creative before becoming a young physical activity leader"**

**The overall % agreement for this item, across all participants was 53%. Considering this, and your stakeholder group's average score, to what extent do you agree or disagree with this statement?**

| 1- Strongly disagree | 2 | 3 | 4 | 5 | 6 | 7 | 8 | 9- Strongly agree |
| --- | --- | --- | --- | --- | --- | --- | --- | --- |
| 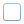 | 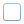 | 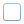 | 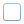 | 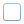 | 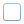 | 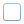 | 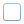 | 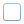 |

**"CV development and support should be embedded within training for young physical activity leaders"**

**The overall % agreement for this item, across all participants was 63%. Considering this, and your stakeholder group's average score, to what extent do you agree or disagree with this statement?**

| 1- Strongly disagree | 2 | 3 | 4 | 5 | 6 | 7 | 8 | 9- Strongly agree |
| --- | --- | --- | --- | --- | --- | --- | --- | --- |
| 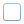 | 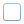 | 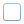 | 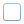 | 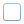 | 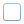 | 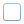 | 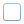 | 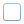 |

**"Learning about the history and rules of specific sports should be embedded within training for young physical activity leaders"**

**The overall % agreement for this item, across all participants was 37%. Considering this, and your stakeholder group's average score, to what extent do you agree or disagree with this statement?**

| 1- Strongly disagree | 2 | 3 | 4 | 5 | 6 | 7 | 8 | 9- Strongly agree |
| --- | --- | --- | --- | --- | --- | --- | --- | --- |
| 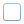 |  |  |  |  |  |  |  |  |
